# Possible relationships of addictive disorders and attention deficit hyperactivity disorder (ADHD)

**DOI:** 10.1192/j.eurpsy.2021.457

**Published:** 2021-08-13

**Authors:** M. Krupa

**Affiliations:** Educational Of Doctoral School, University of Szeged, Szeged, Hungary

**Keywords:** ADHD, adolescents, video game use disorder, addictive disorder

## Abstract

**Introduction:**

One of the most recent topics in addictive disorders is videogame-use disorder which is continuously under research, especially in adolescents. The specific structure of digital games (immortality, infinity, etc.) can sensitize adolescents to the development of problematic use. The number of researches about problematic video game use has increased significantly during the last decade. In 2013, this problem was included among “Disorders requiring further research” in DSM-5, and it was also included in ICD-11 as a separate diagnostic category in 2019.

**Objectives:**

We review studies investigating the association between the co-occurrence of ADHD and video game use in adolescents. We attempt to summarize new theoretical approaches to video game use disorder and the areas of present research.

**Methods:**

We conducted a literature search in 4 databases (PubMed, Medline, Google Scholar, Web of Science) using keywords (ADHD, adolescents, video game use disorder, internet addiction, game addiction) over the past 5 years. Exclusion criteria were the following: publication date before 2014, adult population, or comorbidity beside ADHD.

**Results:**

The comorbidity of video game use disorder and ADHD was frequent. Primarily cross-sectional studies examined the presence of hyperactivity, attention deficit, and impulsivity symptoms separately. The presence of attention deficit clearly showed an association with the development of video game use disorder.

**Conclusions:**

Adolescents diagnosed with ADHD have a greater possibility of developing video game use disorder and/or problematic psychoactive substance users. More attention should be paid to this comorbidity in not only the diagnostic process, but also in the development of prevention programs.

**Disclosure:**

No significant relationships.

